# Yeast Transcription Termination Factor Rtt103 Functions in DNA Damage Response

**DOI:** 10.1371/journal.pone.0031288

**Published:** 2012-02-15

**Authors:** Indukuri Srividya, Sirupangi Tirupataiah, Krishnaveni Mishra

**Affiliations:** Department of Biochemistry, School of Life Sciences, University of Hyderabad, Hyderabad, India; Tulane University Health Sciences Center, United States of America

## Abstract

YKu70/YKu80 is a heterodimer that is essential for repair of DNA double strand breaks through non-homologous end joining pathway in the yeast *Saccharomyces cerevisiae*. Yku70/80 proteins are associated with telomeres and are important for maintaining the integrity of telomeres. These proteins protect telomeres from recombination events, nuclease attacks, support the formation of heterochromatin at telomeres and anchor telomeres to the nuclear periphery. To identify components in molecular networks involved in the multiple functions of Yku70/80 complex, we performed a genetic screen for suppressors of *yku70* deletion. One of the suppressors identified was *RTT103*, which encodes a protein implicated in transcription termination. We show that *rtt103*Δ are sensitive to multiple forms of genome insults and that *RTT103* is essential for recovery from DNA double strand breaks in the chromosome. We further show that Rtt103 associates with sites of DNA breaks and hence is likely to play a direct role in response to DNA damage.

## Introduction

Genomes are constantly subjected to multiple forms of damage and if left unattended, can lead to mutations and chromosomal aberrations that result in cell death and diseases like cancer. Consequently, cells have evolved several mechanisms to detect and repair damage to the genome. Damage to DNA induces a DNA damage response (DDR) that essentially comprises of three arms. The first of these is the cell-cycle checkpoint that arrests the cell cycle in order to prevent damaged DNA from being replicated or transmitted; second, the DNA repair pathways that repair the break and lastly an apoptotic pathway leading to death of the cell if the damage is irreversible. DNA damage is generally induced by exposure to UV irradiation, genotoxic drugs, ionising radiation or other metabolites that generate reactive oxygen species. Depending on the type of lesion and the stage of cell cycle, different repair mechanisms may be activated. These pathways of repair and checkpoint mediated cell cycle arrest are conserved across eukaryotes, reviewed in [1], [2].

A particularly dangerous form of damage is the DNA double-strand break (DSB). These may arise due to, among other factors, exposure of DNA to ionising radiations or genotoxic drugs. Cells repair this form of DNA damage either through non-homologous end joining (NHEJ) or through homology-mediated repair. In brief, during homology-mediated repair, such DNA lesions are recognized by the MRX complex (Mre11-Rad50-Xrs2) in budding yeast, *S. cerevisiae*. This in turn recruits Tel1, a DNA-dependent protein kinase that phosphorylates multiple substrates including histone H2A, which recruits repair proteins. Tel1 also phosphorylates Rad53, a protein kinase that activates Dun1, which in turn leads to induction of transcription of DNA damage inducible genes. Rad53 also induces cell cycle arrest through phosphorylation of other substrates like Cdc5 and Cdc20. The single stranded DNA generated at the breaks recruits a different set of proteins including Rpa1, that recruit another DNA-dependent protein kinase Mec1 which again induces the phosphorylation of Rad53 kinase via Rad9 kinase, leading to cell cycle arrest and transcriptional up regulation of the DDR genes (reviewed in [1]).

Although a lot is known about the different pathways that operate in repair of DNA lesions and the various checkpoint proteins that together contribute to cell cycle arrest and repair of damage, it is increasingly apparent that there are large gaps in our knowledge about the large number of cellular processes are actually required for recovery from damage. Multiple screens have been done to identify components that affect genome stability; each new screen has revealed new proteins and pathways that are involved in the response to DNA damage. Approaches involving the genome-wide measurement of transcriptional responses to DNA damage by UV or MMS show changes in transcriptional status of over 25% of the genome [3], [4], [5]. In competitive fitness assays using the whole genome knock-out strains of yeast, several pathways including those involved in ubiquitination, gene silencing, and transport across the mitochondrial membrane were identified [6]. Similarly in protein localization-based screens that look at the key damage sensors like H2Ax in mammals [7] or Rad52 in yeast [8], pathways involving nuclear transport, RNA processing, protein modification and chromosomal structural proteins were discovered. How all these responses contribute to recovery from DNA damage needs to be elucidated. An important outcome of these studies is the realization that even though there are a few core damage response genes and repair pathways that do appear in most screens, a large number of new and previously unidentified genes and molecular networks are discovered with each new screen.

In this work, we discovered that a transcription termination factor *RTT103* (Regulator of Ty1 transposition) is critical for maintaining genome integrity. This work was initiated to derive insights into the function of Yku70/80 heterodimer in genome integrity. Yku70/80 is a DNA binding heterodimer consisting of Yku70 and Yku80 proteins, that are conserved from yeast to mammals [9]. They play a major role in repair of DNA breaks by NHEJ. Additionally, Yku70/80 are important for many telomeric functions, including loading of telomerase to telomeres, protecting telomeres from nucleolytic digestions, establishing stable silent chromatin at telomeres and also in anchoring telomeres to the nuclear periphery [10], [11], [12], [13], [14], [15]. *yku70/80* mutants are temperature sensitive and die at 37°C with enlarged budded cells that contain more than G2 DNA content [16], [17].

In order to isolate interacting partners that contribute to the multiple roles of Yku proteins, we carried out a multicopy suppressor screen for temperature sensitivity of *yku70*Δ. In this process, we isolated *RTT103* as a partial suppressor of temperature sensitivity. We show that *rtt103*Δ are severely defective in repairing DNA breaks although they do not affect signalling of DNA damage or repair of non-chromosomal substrates by NHEJ. Further, we show that Rtt103 protein physically associates with the sites of DNA breaks and hence is likely to play a direct role in the repair of DNA breaks. This unanticipated role for Rtt103 in DNA repair adds it to the list of RNA processing factors like CSTf [18] and Sen1 [19] that have been recently shown to have roles in genome stability.

## Materials and Methods

### Yeast strains and plasmids

All strains used in this study were isogenic with W303 background (*RAD5*+) unless otherwise indicated. The genotypes of strains used are described in Table 1. Manipulations in the yeast strains were carried out by PCR based homologous recombination using vectors and methods described [20]. Gene manipulations were confirmed by Southern blots. All mutations used are gene replacements unless specified otherwise. Multicopy *RTT103* plasmid (CKM233) was constructed by digesting the KM93 plasmid with *Hpa*I and *Nsi*I, and ligating to YEplac181 vector digested with *Pst*I and *Sma*I . Single copy *RTT103* was constructed by digesting the CKM233 plasmid with *Kpn*I and *Sph*1 and ligating to YCplac22 digested with *Kpn*I and *Sph*1. pCH10 plasmid which contains *RAD53-9myc* in YCplac111 was from Marco Foiani [21].

**Table 1:** List of the yeast strains used in this study.

Name	Genotype	Source/Reference
KRY2	MATa *leu2-3,112 his3-11,15 ura3-1ade2-1 trp1-1 can1-100*	Rodney Rothstein
KRY3	MA α *leu2-3,112 his3-11,15 ura3-1ade2-1 trp1-1 can1-100*	Rodney Rothstein
KRY105	MATa *adh4::ADE2* TEL VII L	This Study
KRY171	KRY105 except *yku70::KanMx*	This Study
KRY172	KRY 193 except MATα *yku70::KanMx*	This Study
KRY193	MATa *adh4::URA3* TEL VII L	This Study
KRY230	KRY105 except MATα *rtt103::KanMx*	This Study
KRY290	KRY 105 except MATα *rtt103::KanMx*	This Study
KRY285	KRY 193 except MATα *rtt103::KanMx*	This Study
KRY286	KRY 193 except *rtt103::KanMx yku70::LEU2*	This Study
KRY290	KRY 105 except MATα *rtt103::KanMx yku70::LEU2*	This Study
KRY371	MATa Δ *lys2::pGAL-ISCEI ISceI::URA3::ISceI*	This study
KRY375	KRY 371 except *rtt103::KanMx*	This Study
KRY376	KRY371 except *yku70::LEU2*	This Study
KRY379a	KRY371 *rtt103::KanMx yku70::LEU2*	This Study
KRY448	KRY371 except *RTT103* 13Myc	This Study
KRY482	W303 *rtt103* diploid	This Study
KRY473	W303 MATa *rad1::LEU2*	Hannah Klein
KRY631	MATα *RAI1 his3Δ1 leu2Δ0 ura3Δ0* (BY4739)	Arlen Johnson
KRY632	KRY 631 except *rai1::KamMX*	Arlen Johnson
KRY633	MATa *ura3-52 leu2Δ1 trp1Δ63* (FY23)	Arlen Johnson
KRY634	KRY 633 except MATα *rat1-1 ts*	Arlen Johnson
KRY615	Mat a *ade2-1 trp1, his3, ura3 can1-100 leu2-k::ADE2 URA3::leu2-k rad5-535*	Andre Aguilera
KRY650	MATα *ade2-1 trp1, his3, ura3 can1-100 leu2-k::ADE2-URA3::leu2-k rtt103::KanMx rad5-535*	This study
KRY652	MATα *ade2-1 trp1, his3, ura3 can1-100 leu2-k::ADE2-URA3::leu2-k hpr1Δ3::HIS3 rad5-535*	Andre Aguilera
KRY622	MATα pGAL*HO::LEU2 rad5-535*	David Shore
KRY624	MATa pGAL*HO::LEU2 rai1::KanMx rad5-535*	This study
KRY646	MATa pGAL*HO::LEU2 rtt103::KanMx rad5-535*	This study
KRY443	KRY105except *mec1Δ::TRP1, sml1Δ:: HIS3*	This study
KRY445	KRY105except MATα *rtt103::KanMx*	This study

### Western blot analysis

To examine phosphorylation of Rad53, cells carrying *RAD53-9myc* were treated with 0.02% MMS for 2 hrs or left untreated and total protein extracted using TCA [21]. Briefly yeast extracts were prepared by glass bead beating in 20% trichloroacetic acid (TCA), washing the glass beads in 5% TCA and combining the wash with the lysate. The protein suspension was pelleted, resuspended in 1× Laemmli loading buffer (pH 8.0), boiled for 2 min, pelleted, and the supernatant was used for Western blots. Protein was run on 8% SDS-PAGE gels and transferred to PVDF membrane. Anti-myc staining was done with 9E10 (BabCo) antibody. Same blots were probed with Sir2p antibody (Santa Cruz) for loading control.

### DNA Damage Assays

#### Plasmid rejoining assay

pRS313 DNA (100 µg) was digested with EcoRI to completion. This linearised DNA was then used to transform yeast by the lithium acetate method. Parallel transformations with the same competent cells were performed with an equivalent amount of uncut plasmid to enable normalisation for minor differences in transformation efficiencies between strains and between experiments. Following transformation, cells were plated and colonies arising on selective media (SC-HIS) after 3–4 days were counted. We typically obtained 1600 to 2200 transformants with the supercoiled plasmid in all strains; the linearized plasmid recovery was in the range of 1100 to 1500 in wildtype and *rtt103*Δ, while in *yku70*Δ we obtained around 40 colonies.

#### MMS assay

Yeast strains were grown overnight to mid-log phase in YPD medium. 10-fold serial dilutions of the strains were done and 5 µl of the sample was spotted on YPD plates and YPD containing different concentrations of MMS. The plates were incubated for 2–3 days at 30°C.

#### I-SceI endonuclease assay

For I-SceI endonuclease assay, KRY304 strain was used which has two I-SceI sites inserted in opposite orientations on each side of the *URA3* gene on chromosome V. The sequence encoding the nuclease was inserted at a different locus and placed under the control of a galactose-inducible promoter. Strains were grown overnight and spread on SC-GLU and SC-GAL plates. Colonies were counted after incubation at 30°C for 4 days.

#### Chromosome hyper-recombination assays

The strains used for this study have *ADE2* and *URA3* genes flanked by *leu2-k* repeats [22]. Recombination between the *leu2-k* repeats results in loss of *ADE2* and *URA3*. The number of recombinants was calculated by counting the number of colonies on SC+ FOA plates, which indicate the loss of *URA3*; recombination was confirmed by the appearance of red colonies showing loss of *ADE2*. Three independent segregants were used for quantification of recombination.

#### Chromatin Immunoprecipitation

ChIP experiments were done by following the method described [23]. Yeast strain KRY448 was grown in 50 ml of SC broth till OD600 reached 0.5–1.0. These cultures were cross-linked with formaldehyde and cross links were quenched with glycine. Cells were pelleted, washed with ice cold TBS buffer, and lysed with ice cold lysis buffer containing protease inhibitor. Lysates were prepared with glass beads and were then sonicated in Biorupter Sonifier at power setting of 15 sec pulse on and 2 min off to shear the chromatin to average length of 500 bp . The supernatant was precleared by adding 30 µl bed volume of Protein A Sepharose beads (Amersham Biosciences) and incubated for 1 hr at 4°C with constant rotation. Samples were centrifuged and 50 µl of sample was taken as input DNA. 1 µl of primary antibody against myc epitope of Rtt103p (Abcam) was added to the sample and incubated for overnight at 4°C with constant rotation. 30 µl of Protein A Sepharose beads were added to the chromatin antibody mix, and incubated for 2 hr at 4°C with constant rotation. Chromatin imunoprecipitate was eluted first with 1% SDS in TE and then with 150 µl of 0.67% SDS in TE buffer by incubating at 65°C for 10 min. DNA from bound and unbound chromatin (input sample) was purified by phenol:chloroform extraction and ethanol precipitation. DNA sample from ChIP experiments were analysed by Real-Time PCR using SYBER green master mix according to manufacturer's instructions on an Applied Biosystems 7500 HT fast Real-Time PCR system. Primers for real-time PCR were designed by taking sequences at different regions near the *URA3* gene flanked by I-Sce1 sites on chromosome V. Primer sequence information can be provided upon request. Relative quantification of immunoprecipitated DNA was done based on comparative C_t_ value method using sequence detection software.

#### Northern blots

Northern blots were done as described in [24]. RNA was extracted using Ambion RNA isolation kit. For Northern blots, 10 µg of RNA was loaded onto 1% formaldehyde agarose gels, separated by electrophoresis, and then transferred to N+ membranes. Blots were blocked for 1 hr at 55°C and then incubated with ^32^P-labeled DNA probes. Hybridizations were performed for 16 hr at 55°C.

#### Microscopy

For sporulation experiments, cultures were fixed with ethanol and stained with DAPI. Immunofluorescence experiments were performed as described in [25]. For localization of Rtt103, antibodies to myc epitope and Nsp1 were from Abcam. AlexaFluor conjugated anti mouse and anti rabbit antibodies were from Molecular Probes. Olympus inverted microscope IX81 was used to visualize and capture images.

## Results

### Multiple copies of *RTT103* suppress the temperature and DNA damage sensitivity of *yku70* mutants

*yku70/80* mutants are sensitive to temperature; they grow slowly at 35°C and die at 37°C. In order to characterize the temperature sensitivity of *yku70*Δ, we performed a multicopy suppressor screen to identify processes that are compromised at 35°C. *yku70*Δ (KRY172) were transformed with a genomic library in a high copy number vector (gift from K. Nasmyth) and plated at 35°C and incubated for 2 days. Any large colony that appeared in two days was isolated and rechecked for improved growth at 35°C and 37°C. The plasmids were isolated and retransformed into *yku70*Δ and tested again for growth at elevated temperatures.

**Figure 1:**
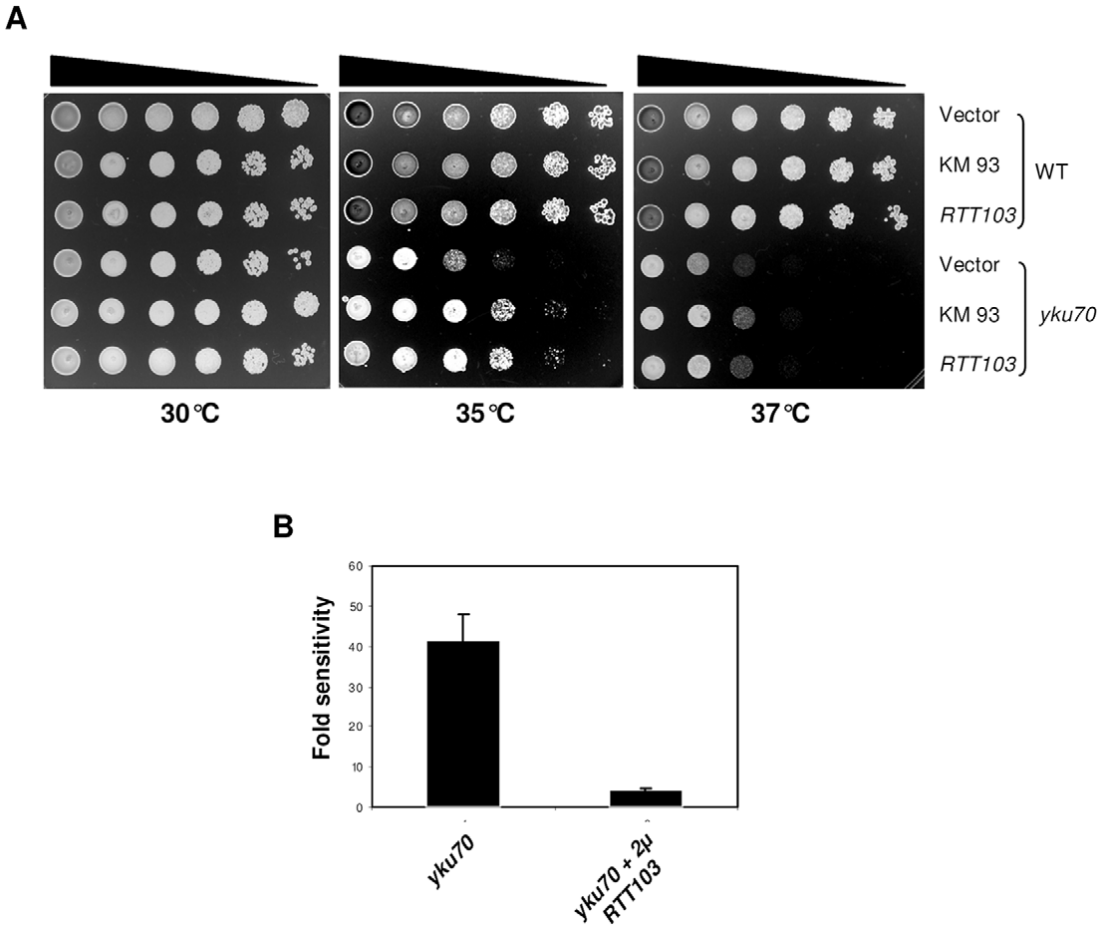
*RTT103* partially suppresses the *yku70* ts phenotype. WT (KRY193) and *yku70* (KRY172) mutants were transformed with empty vector, KM93 and *RTT103.* 5 µl of 10-fold serial dilutions of yeast cultures were plated on SC-LEU plates and incubated at 30°C, 35°C and 37°C for 2–3 days. (**1b**) Quantification of temperature sensitivity. The temperature sensitivity of *yku70* was quantified by plating out appropriate dilutions of 3 independent cultures at the appropriate temperatures. Sensitivity of WT was set to 1. *yku70* is approximately 40 fold sensitive and upon overexpression of *RTT103*, the sensitivity to temperature is reduced by approximately 7 to 8 fold. Error bars indicate SD.

Two plasmids that reproducibly improved growth were obtained in this screen; one of the plasmids (KM95), showed strong suppression of *yku70* phenotype and upon sequencing was found to contain the full length *YKU70* gene. This *YKU70* gene complemented the *yku70* phenotype (not shown). The second plasmid (KM93) that partially suppressed the *yku70* temperature sensitivity phenotype contained the complete sequence of the *RTT103* gene. *RTT103* was subcloned in YEplac181 vector and was tested to see if it suppressed *yku70* phenotype (Figure 1a). As shown in Figure 1a, *yku70*Δ is lethal at 37°C and partially defective for growth at 35°C. Upon over-expression of KM93 or just *RTT103* on a multicopy plasmid, *yku70*Δ grew better at 35°C (compare row 4 with rows 5 and 6). As extent of suppression of temperature sensitivity was modest, we quantified the phenotype by plating cells at different temperatures. As shown in Figure 1b, we found that elevated dosage of *RTT103* improved growth of *yku70*Δ by 7 to 8-fold. However, *RTT103* could not suppress lethality at 37°C. Therefore, we conclude that an elevated dose of *RTT103* partially suppresses the temperature sensitivity of *yku70* mutants.

**Figure 2:**
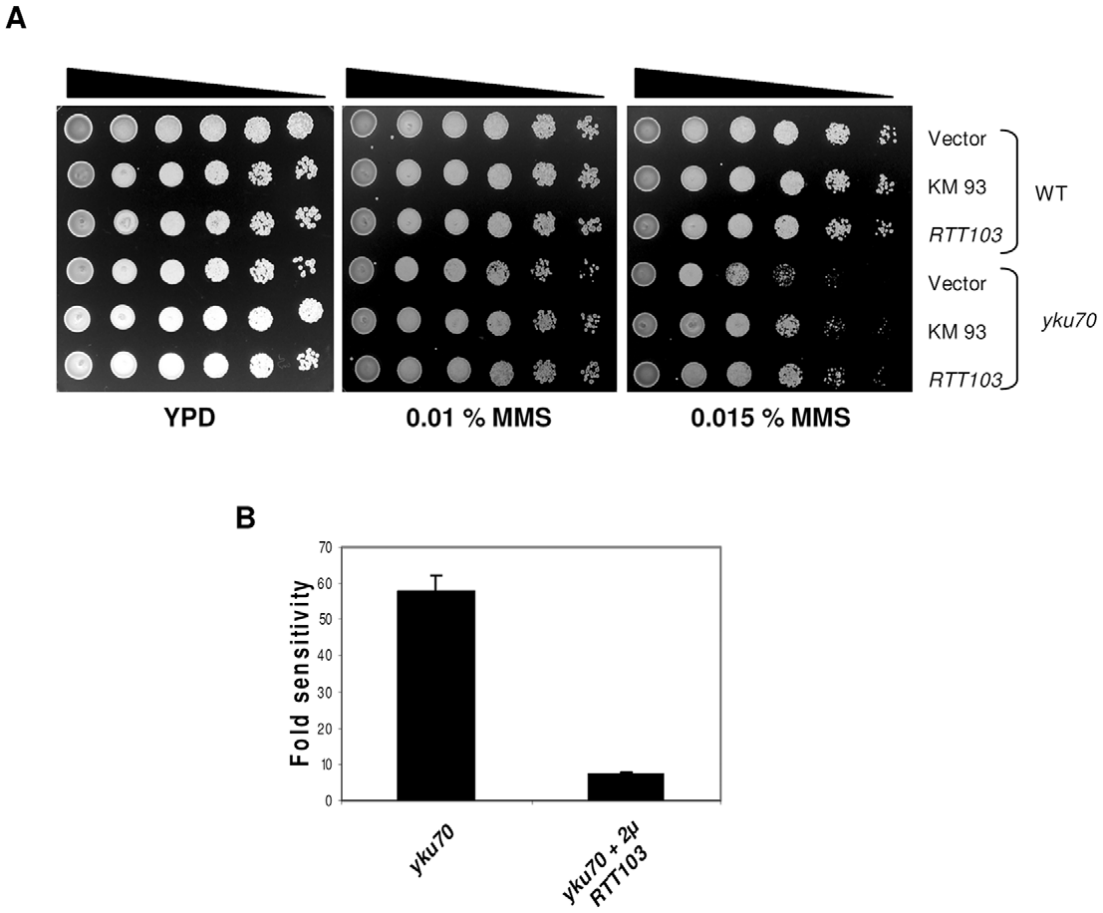
*RTT103* partially suppresses the the MMS sensitivity of *yku70.* WT (KRY193) and *yku70* (KRY 172) mutants were transformed with empty vector, KM93 and *RTT103.* 5 µl of 10-fold serial dilutions of yeast cultures were plated on SC-LEU plates containing MMS and incubated at 30°C for 2–3 days. (**2b**) Quantification of MMS sensitivity. The MMS sensitivity of *yku70* was quantified by plating appropriate dilutions on plates with and without MMS. Sensitivity of WT was set to 1. *yku70* is approximately 60 fold sensitive and upon overexpression of *RTT103* the sensitivity to MMS is reduced by approximately 8 fold. Quantification was done for three independent cultures and error bars show SD.

Since *YKU70* is involved in several processes including DNA repair, telomere metabolism and gene silencing we tested if multiple copies of *RTT103* also suppressed any of these phenotypes. We found that while neither gene silencing nor telomere length defects of *yku70* mutants were affected by *RTT103* (data not shown), the DNA repair phenotype was partially suppressed by over-expression of *RTT103* (Figure 2). *yku70/80* mutants are sensitive to MMS, an alkylating agent, which produces DSB during repair. Therefore, we plated wild type and *yku70*Δ transformed with either empty vector or *RTT103* on plates containing MMS. As shown in Figure 2a and Figure 2b, *yku70* mutants are over 50-fold more sensitive to MMS than wild type (row 4). Upon elevated dosage of *RTT103*, there is a marked improvement in survival, and these cells are only 7-fold more sensitive than wild type, suggesting that *RTT103* on multicopy plasmid partially suppresses the *yku70* temperature sensitivity and MMS sensitivity. *RTT103* on single copy plasmid could not suppress these phenotypes indicating that suppression requires multiple copies of *RTT103* (data not shown).

### *rtt103* deletion enhances *yku70* sensitivity to temperature and are sensitive to MMS

**Figure 3:**
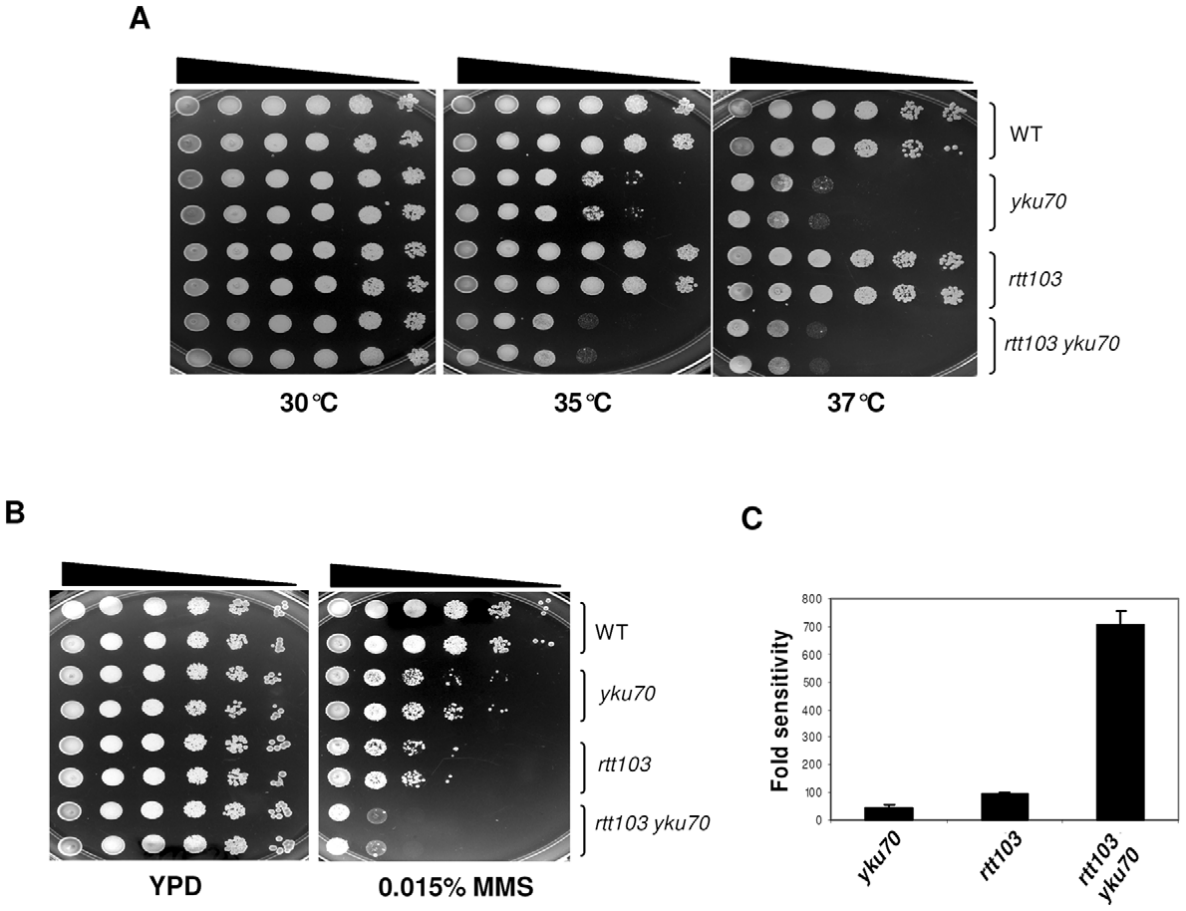
*rtt103*Δ are MMS sensitive and enhance the temperature sensitivity and MMS sensitivity of *yku70* mutation. 5 ul of ten-fold serial dilutions of wild type (KRY193), *yku70* (KRY172), *rtt103* (KRY285) and *yku70rtt103* (KRY286) double mutants were spotted on YPD plates and incubated at 30°C, 35°C and 37°C (**3a**) or on plates containing MMS (**3b**) for 2 to 3 day**s**. Two independent cultures for each mutant are shown here. *rtt103*Δ are sensitive to MMS and enhance both the temperature sensitivity and MMS sensitivity of *yku70*Δ. (**3c**) Quantification of MMS sensitivity. The MMS sensitivity of *yku70*, *rtt103*, *rtt103 yku70* was quantified as described in Figure 2. The sensitivity to MMS for *yku70*, *rtt103* and *rtt103 yku70* were approximately 55, 100 and 750 fold respectively. Values plotted are from three independent cultures and error bars show SD.

*RTT103* was initially isolated in a screen for mutants that enhance the transposition of Ty1 elements [26]. It was later shown to participate in transcription termination [27]. Additionally, *rtt10*Δ were also reported to increase the number of Rad52 foci [8]. Since elevated *RTT103* dosage could suppress *yku70* mutant sensitivity to DNA damage, we investigated if *RTT103* is required for DNA repair. To this end we generated *rtt103*Δ by replacing the complete *RTT103* ORF with *KanMx* (KRY285). The *rtt103*Δ were tested for sensitivity to temperature. As shown in Figure 3a and as expected, growth of *yku70*Δ was arrested at 37°C and impaired at 35°C. *rtt103*Δ were not sensitive to increased temperatures; growth was comparable to that of wild type strains. However, the *yku70rtt103* double deletions (rows 7, 8) showed increased sensitivity to temperature, as reflected by poor growth at 35°C when compared to *yku70*Δ. Thus, *rtt10*Δ exacerbates the temperature sensitivity of *yku70*Δ.

We next checked the effect of *rtt103*Δ on DNA repair. First, we tested sensitivity of the cells to MMS. Serially diluted cultures of wild type, *yku70*Δ, *rtt103*Δ and *yku70rtt103* were plated on normal YPD or YPD containing MMS. As shown in Figure 3b, *rtt103*Δ were sensitive to MMS, as much as or more than *yku70*Δ (compare rows 3, 4 to rows 5, 6). The double deletions were even more sensitive to MMS than either single mutant (row 7,8). These phenotypes were quantified by plating out three independent cultures: *yku70*Δ single mutants were 55-fold more sensitive than wild type, the *rtt103*Δ were 100-fold more sensitive, and the double mutants were over 700-fold more sensitive. These data indicate that *rtt103* enhances the *yku70* defective phenotypes in a synergistic manner.

### *rtt103*Δ are unable to recover from DNA double strand breaks

**Figure 4:**
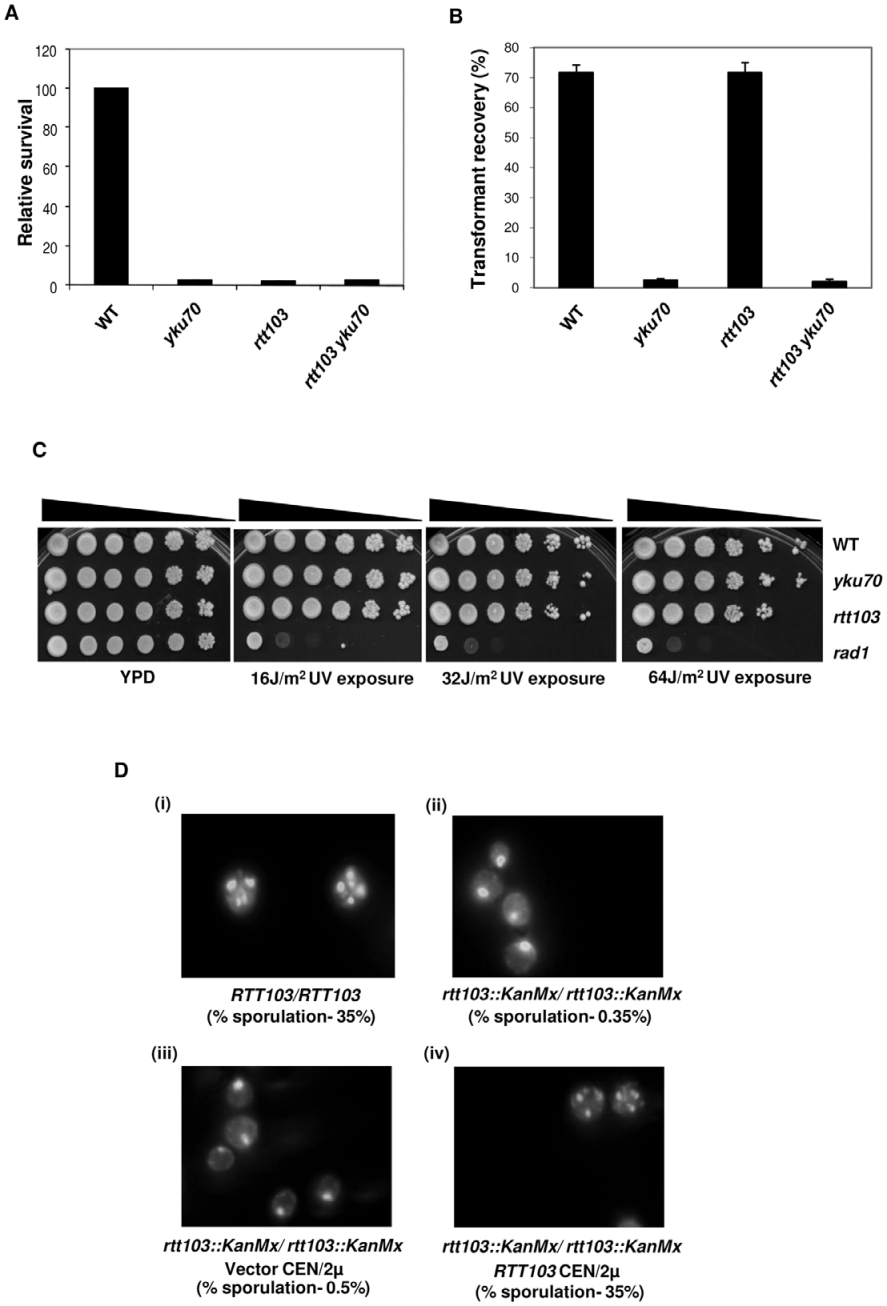
*rtt103*Δ are sensitive to various kinds of DNA damage. (**4a**) *rtt103*Δ are sensitive to *Sce*-I endonuclease. Strains of WT (KRY304), *yku70* (KRY376), *rtt103* (KRY375) and *rtt103 yku70* (KRY379) with two *Sce*-I sites on either side of the *URA3* gene were induced with galactose to produce DSBs. The relative survival on galactose versus glucose was calculated from three independent cultures for each strain and error bars show SD. (**4b**) WT (KRY105), *yku70* (KRY171), *rtt103* (KRY230) and *rtt103yku70* (KRY290) strains were transformed with supercoiled or linearized pRS313. The transformants were plated on SC-HIS plates in duplicates and incubated at 30°C for 2–3 days. The value plotted is the percentage of linear plasmid recovered relative to supercoiled plasmid for each strain from three independent transformation experiments.. (**4c**) Wild type (KRY105), *yku70* (KRY171), *rtt103* (KRY230) and *rad1* (KRY473) strains were grown to mid log phase, 10-fold serially diluted and spotted on YPD plates. They were then exposed to UV radiation and incubated at 30°C for 2 days. (**4d**) *rtt103*Δ homozygous strains are severely defective in sporulation (**i**) and (**ii**) WT and *rtt103* homozygous diploids were incubated on YPK plates for induction of sporulation and stained with DAPI to visualize nuclei after 4 days. *rtt103*Δ were transformed with either empty vector (**iii**) or with single copy of *RTT103* (**iv**) and incubated on YPK plate for 4 days. DAPI images from this stage are shown. Quantification of spores was done by scoring 500 to 4000 cells.

As MMS generates multiple DNA breaks all over the genome, and could potentially affect several genome functions, we tested the role of *RTT103* when single or two DSBs were introduced specifically. For this, we used a strain where two sites recognized by I-SceI endonuclease have been introduced bracketing *URA3* gene. This strain also contains an insertion of galactose inducible I-SceI endonuclease gene in the genome [28]. We generated *yku70* and *rtt103* deletions in this strain and tested the efficiency of colony formation on galactose plates. When I-SceI endonuclease is induced by galactose, DSBs are generated on either side of *URA*3 gene. Wild type cells can repair the breaks and form colonies (Figure 4a). Strains that cannot repair the DNA break are expected to be growth arrested and unable to form colonies on galactose plates. We plated wild type, *yku70*Δ, *rtt103*Δ and *yku70rtt103* deletions carrying the I-SceI sites and endonuclease on plates containing galactose. Numbers of full sized colonies that appeared after 3 days were counted. As shown in Figure 4a, *yku70*, *rtt103* and *yku70rtt103* deletions were all severely affected and very few colonies could be recovered. However, wild type cells were able to form colonies as they could repair the breaks at the expected frequency [28]. We noticed that even though *rtt103* deletions did not form many full-sized colonies, there were numerous tiny colonies on the plate. This suggests that *rtt103*Δ, unlike *yku70*Δ, underwent a few divisions before they succumbed.

### *RTT103* is not required for plasmid end joining or repair of UV induced damage

As the specific DSB assay indicated that *RTT103* was required to repair these breaks *in vivo*, we directly tested if end joining was affected in *rtt103*Δ by a plasmid rejoining assay. This assay reports the efficiency of end-joining by NHEJ. When linearized CEN plasmids are transformed into yeast, they are recircularized by end joining (most efficient) or are integrated into the genome (less efficient). However, when end-joining is defective, as seen in *yku*70/80 mutants or DNA ligaseIV mutants [29], very few transformants are recovered. Equal amounts of plasmids linearized with *Eco*RI were transformed into wild type, *yku70*Δ, *rtt103*Δ and *yku70rtt103* deletions, and the numbers of transformant colonies were counted. The data are represented as fraction of linear plasmid recovered relative to the supercoiled plasmid. As expected, a high number of transformants were obtained in wild type cells while very few could be recovered in *yku70*Δ (Figure 4b). The number of transformants in *rtt103*Δ were comparable to wild type, suggesting that *RTT103* is not required for end-joining of plasmids.

We then tested if *rtt103*Δ were sensitive to damage by UV irradiation, by comparing them to *rad1*Δ that is extremely sensitive to UV radiation and *yku70*Δ that is not sensitive to UV. We found that *rtt103*Δ were not sensitive to UV radiation (Figure 4c) and were quite indistinguishable from wild type. These results indicate that *RTT103* is required for recovery from MMS- or endonuclease-, but not from UV- induced damage. In sum, the data described above show that *RTT103* is required for repair of chromosomal breaks although could be dispensable for UV damage.

### *rtt103*Δ are defective in sporulation

As all these experiments indicated that *RTT103* is essential for efficient repair of DNA damage, we asked if repair of naturally occurring DSBs also requires *RTT103*. When yeast cells undergo meiosis, an early step in prophase I is the introduction of several DSBs by Spo11: many of these initiate recombination events and others are repaired without recombination. We asked if *RTT103* has any effect on the repair of these breaks. For this, we generated diploid, *rtt103*Δ*/rtt103*Δ cells and plated on potassium acetate plates to induce sporulation. *rtt103/rtt103* diploids were severely defective in sporulation (0.5%; 4 out of 800 cells scored), although the wild type cells cells sporulated efficiently (35%) on the same plates. To confirm this, we stained the cells with DAPI. As shown in Figure 4d, we could clearly see 4 nuclei in many wild type cells but in *rtt103*Δ there were none. However, *rtt103*Δ carrying a copy of *RTT103* on a plasmid could sporulate as efficiently as the wild type cells. We noted that a previous genome-wide screen for meiosis and sporulation reported that *rtt103*Δ sporulated normally [30]. However, we observed that *rtt103*Δ*/rtt103*Δ diploids obtained from BY4741 and BY4742 also were severely defective in sporulation (no spores at all in 4000 cells scored). It is possible that *rtt103* escaped detection in the previous genome-wide study. These data indicate that *RTT103* is essential for successful meiosis, probably because it is essential to repair the DSBs induced during this process.

### DNA damage signalling is intact in *rtt103*Δ

A key difference between the plasmid end-joining assay and the chromosome break assays is the nature of response to damage: when there is a chromosome break, cells arrest their division cycle and signal the presence of the break to the repair proteins. This signal transduction cascade culminates in the recruitment of proteins that execute the repair, and when the repair is complete, the signal is turned off. But in case of plasmids, as the break lies on an extra-chromosomal element and not on the chromosome, there is no cell cycle arrest or activation of the signalling cascade. Because *rtt103*Δ were sensitive to chromosomal breaks but not plasmid breaks, we reasoned that the end-joining process *per se* is not affected, but the signal transduction cascade could be affected in the *rtt103*Δ. In order to test this, we checked the phosphorylation of Rad53, the final effector kinase in the pathway. Rad53 phosphorylation activates the phosphorylation of Dun1 protein by Rad53 kinase; Dun1 activation leads to transcriptional upregulation of a set of repair specific genes. Rad53 is phosphorylated in response to DNA damage primarily by Mec1, and this is facilitated through interactions between Rad9 and Rad53.

**Figure 5:**
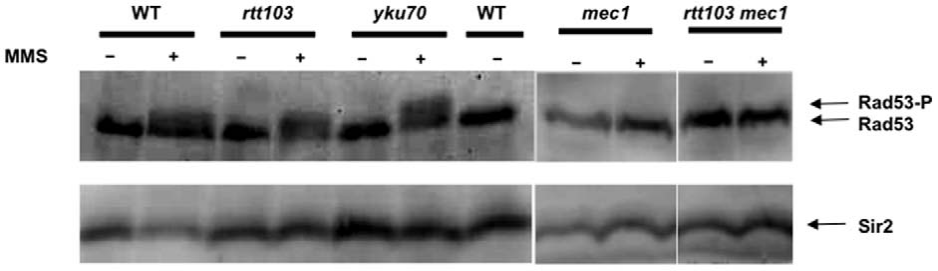
*rtt103*Δ show normal Rad53 phosphorylation. WT, *yku70*, *rtt103*, *mec1* and *rtt103mec1* null strains containing *RAD53-Myc* was treated with MMS for 2 h and anti-Myc western blots were performed. The slower moving fuzzy band indicates phosphorylation of Rad53. Fuzzy bands are visible in all lanes treated with MMS and contain wild type MEC1. *mec1* mutants (lanes 9, 11) do not produce phosphorylated Rad53 upon MMS treatment. Wild type sample was loaded in two lanes of the gel for better comparison of the position of the fuzzy band. Same blot was probed with Sir2 antibody to show that separation of proteins was normal and identical in all lanes.

Wild type, *yku70*Δ and *rtt103*Δ were transformed with plasmid encoding *RAD53-9xMYC* [21] and DNA damage introduced with MMS treatment for 2 hours. Total protein was extracted and Western blots were performed using anti-myc antibody. As shown in Figure 5, the untreated cells show a sharp band at ∼110 kDa. Upon treatment with MMS, we see an upward shift in the Rad53 band, which appears diffuse. This indicates that Rad53 gets phosphorylated upon DNA damage. The shift in molecular weight is same in all three strains, showing that the damage signalling cascade is active in *rtt103*Δ. This phosphorylation was also seen after I-SceI induced damage (data not shown).

We performed two more controls to strengthen this data. First, the same blots were re-probed with antibodies to Sir2 protein to establish that the diffuse bands were not due to abnormal separation of proteins. As shown in the lower panel of Figure 5, Sir2 appeared as a sharp band. Second, we confirmed that the phosphorylation of Rad53 is dependent on DNA-damage signalling by testing the same in a mutant defective in signalling, *mec1* (lanes 8 to 11). No phosphorylation of Rad53 could be detected in *mec1* and *rtt103mec1* double mutants as *MEC1* is required for phosphorylation of Rad53, supporting the hypothesis that the phosphorylation of Rad53 seen in *rtt103*Δ is due to the activation of the signalling cascade.

These experiments showed that the signalling pathway for the damaged DNA is intact in *rtt103*Δ. The downstream effect of this signalling is the transcriptional upregulation of the DNA damage signature genes, namely, *DUN1*, *RAD54*, *RNR2*, *and RAD51*. Their expression is substantially induced in response to both MMS and ionizing radiation [31]. To test if *rtt103*Δ had any defects in the induction of transcription, Northern blots were done to assess RNA levels of *RNR2*, RAD51, *DUN1* and *PLM2* genes in wild type and *rtt103*Δ under normal condition and after DNA damage (Figure S1). We found, as expected, that these genes were induced upon DNA damage and that there was no difference in the levels of RNA induced in wild type and *rtt103*Δ. The result with these representative genes indicates that the defect in *rtt103*Δ is unlikely to affect the expression levels of all DDR genes. However, it is possible that expression of other untested genes are affected by *rtt103*Δ, since previous genome-wide transcriptional studies have reported transcriptional induction of several genes. These data indicate that the DNA damage is sensed and the core downstream response is activated in a normal manner in *rtt103*Δ.

### Transcription termination factors show differential response to DNA repair

**Figure 6:**
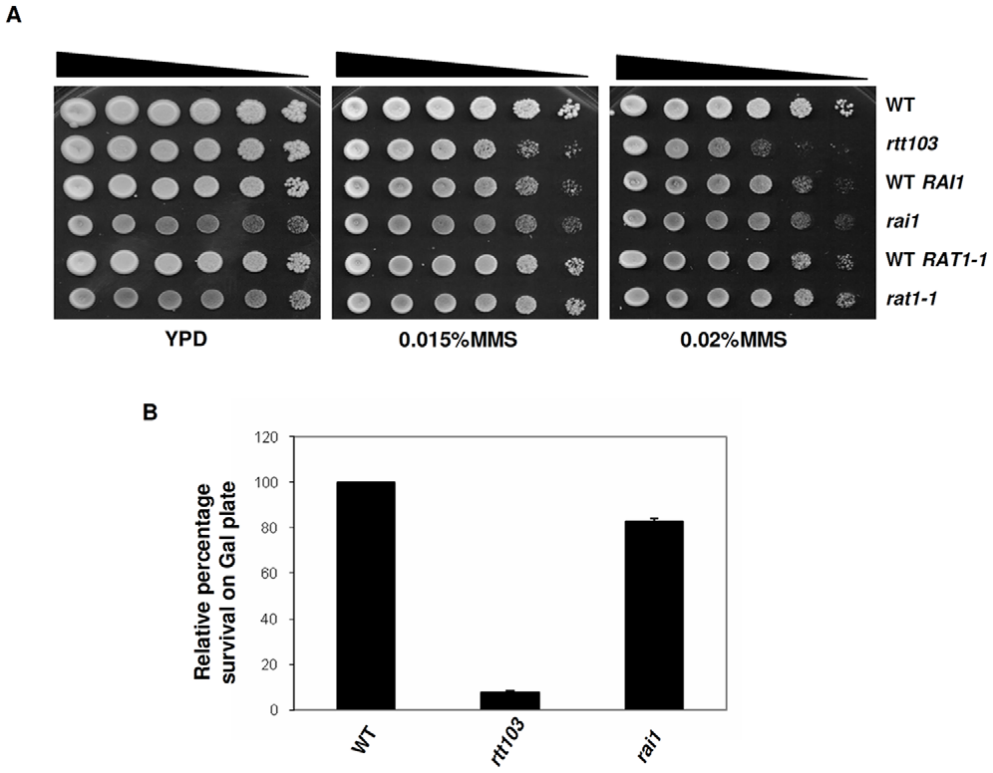
(**6a**) *rat1-1* and *rai1* mutants are not sensitive to MMS. WT (KRY105), *rtt103* (KRY230) WT *RAI1* (KRY631), *rai1* (KRY632), WT *RAT1* (KRY633) and *rat1-1* (KRY634) strains were grown to mid-log phase. 5 µl of 10-fold serial dilutions of yeast cultures were plated on YPD plates containing MMS and incubated at 30°C for 2–3 days. (**6b**) *rai1* mutants are not sensitive to HO endonuclease. WT (KRY622), *rtt103* (KRY646), *rai1* (KRY624), were induced with galactose for HO endonuclease to produce DSBs. The percentage survival on galactose compared to WT was calculated from three independent experiments and error bars denote SD.

Rtt103p copurifies with Rat1 and Rai1 proteins and is synthetically lethal with *rai1* [27]. Rat1, Rai1, and Rtt103 proteins were found to crosslink very strongly at the 3′ends of the genes predicting their involvement in transcription termination. Since Rat1, Rai1, and Rtt103 proteins act in a complex during transcription termination, we wanted to test if defects in *RAT1* and *RAI1* genes show any DNA damage sensitivity when exposed to DNA damaging agents. As shown in Figure 6a, growth of *rtt103*Δ was inhibited by MMS, whereas, growth of *rat1-1* and *rai1*Δ was comparable to the corresponding wild type strains. To further investigate if these factors were required when specific DSBs were introduced, we generated *rai1*Δ and *rtt103*Δ in a strain carrying galactose inducible HO endonuclease that generates a single break in the MAT locus. As shown in Figure 6b, although *rtt10*3Δ are very sensitive to endonuclease induction, *rai1* were not. These results indicate that transcription termination *per se* is not responsible for sensitivity of *rtt103*Δ to DNA breaks and suggest a unique, or at least a more prominent, role for *RTT103* in maintaining genome stability.

### *rtt103*Δ do not enhance chromosomal recombination events

**Figure 7:**
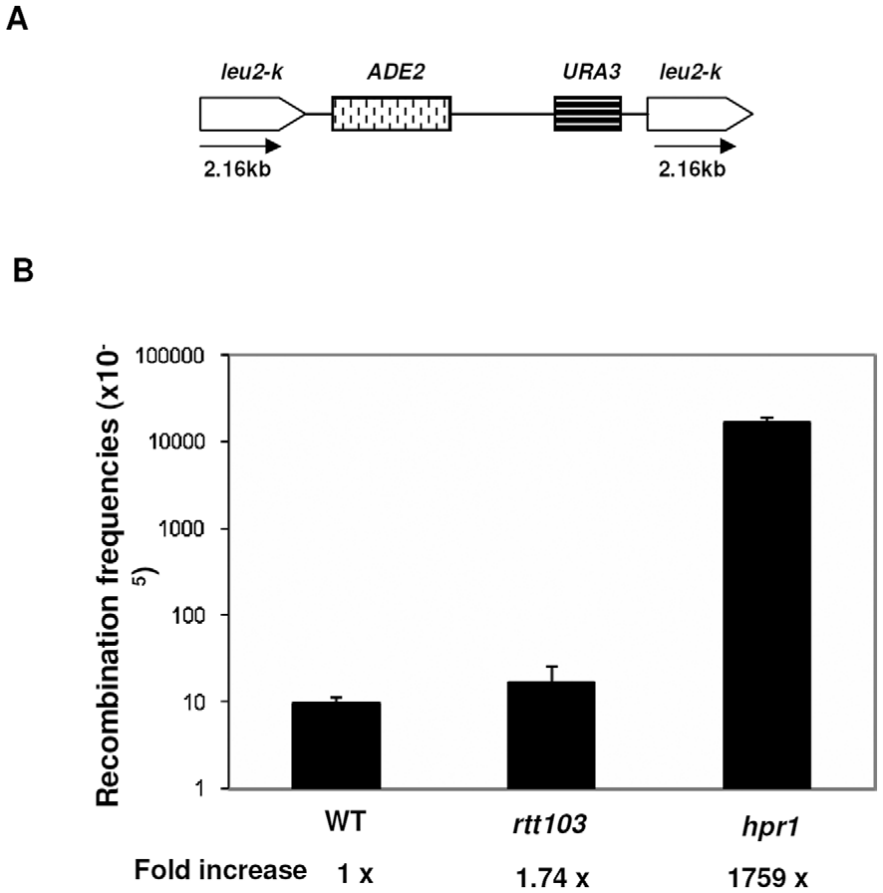
*rtt103*Δ do not show hyper-recombination phenotype. (**7a**) Schematic representation of chromosomal region containing *leu2-k* repeats flanking the *ADE2* and *URA3* genes. (**7b**) WT (KRY615), *rtt103* (KRY650), *hpr1* (KRY652), strains containing the *ADE2*, *URA3* genes flanked by *leu2-k* repeats were plated on SC plates (viability) and SC+FOA plates (recombinants). Recombination between the *leu2* repeats leads to loss of *ADE2* and *URA3* genes. The recombination frequency was determined for three independent segregants and error bars denote SD.

Connections between transcription and genome instability have been well established by studying mutants in the multiple processes that ultimately lead to the accumulation of mature RNA in the cytoplasm [32]. For example, mutations in the components of the THO complex, consisting of Tho2, Hpr1, Mft1 and Thp1, lead to transcription associated hyper-recombination phenotype [33]. Recently, this has been shown for a transcription termination factor in yeast and humans, Sen1 [19], [34]. Sen1 prevents transcription-associated recombination by restricting the occurrence of RNA∶DNA hybrids that might occur during transcription. A hallmark of THO complex mutants and *sen1-1* is the increased chromosomal instability that results from promoting recombination between direct repeats. We tested if *rtt103*Δ also showed similar hyper-recombination phenotypes in the assay described previously [22]. *rtt103*, *yku70* and *hpr1* deletions were generated in a strain carrying *leu2-k::URA3-ADE2:: leu2-k* direct repeat system (Figure 7a) and recombination was tested in wild type and mutants (Figure 7b). As expected, wild type cells had low levels of recombination and *hpr1* had more than 1000-fold increase in recombination. However, *rtt103* had recombination frequencies indistinguishable from wild type cells. These results show that *rtt103* do not stimulate hyper-recombination between repeats. As the *sen1-1* mutant showed increased hyper-recombination, we conclude that Rtt103 is unlikely to function in the same pathway as Sen1 to prevent genome instability. Additionally, *sen1-1* shows synthetic genetic interactions with genes involved in the homologous repair pathway and none with genes involved in NHEJ. However, *rtt103* appears to have the opposite mode of action, by showing synthetic phenotype of increased sensitivity to DNA damage with *yku70*Δ as described in the earlier sections. We also tested the *rtt103rad52* double deletions for damage sensitivity and found that they did not show increased sensitivity (Figure S2). Taken together these data indicate that although both *rtt103* and *sen1* affect genome stability, their mechanisms might be distinct.

### Rtt103 associates with sites of DNA damage

**Figure 8:**
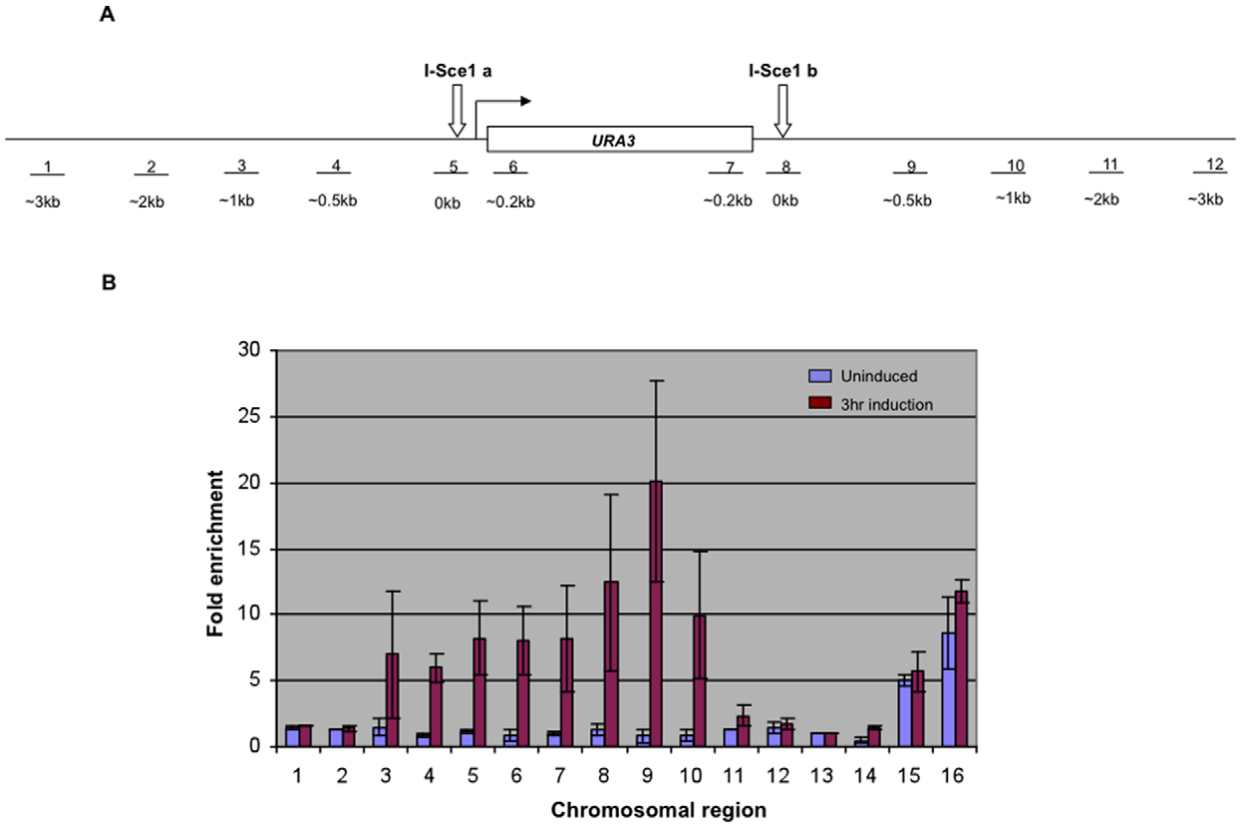
Binding of Rtt103p to the site of DNA damage. (**8a**) Schematic diagram of the region around *URA3* with two flanking I-SceI sites. Bars (1–12) represent the regions up to 3 kb away from I-SceI sites, at which Rtt103p binding was tested (**8b**) ChIP experiment to show Rtt103p binding at the site of damage. Rtt103Myc strain (KRY 448) was grown in galactose medium for three hours and then cross-linked with formaldehyde and followed by immunoprecipitation with anti-myc antibody. X-axis indicates the loci tested (see schematic) and y-axis shows the fold change of Rtt103p binding compared to *SPS2* internal control; blue bars represent Rtt103p association just prior to galactose induction (no cut) and red bars represent association 3 hours after induction. *SPS2* (13) and a region 10 kb from the telomere VI R (14) are negative controls. *PMA1* 3′ region (15) and *ADH1* 3′ region (16) where Rtt103 is reported to crosslink heavily are used as positive controls. Error bars denote SD of three independent immunoprecipitation experiments.

The results shown above indicated that Rtt103 is critical for DNA repair but did not follow the same pathways as Sen1, the other transcription termination factor shown to affect genome stability. In order to gain some clues to its possible mechanisms, we tested if Rtt103 associates with the sites of damage. Rtt103 was first tagged with 13×MYC. It produced an approximately 75 kDa protein and localized to the nucleus. Cells expressing this myc-tagged Rtt103 showed wild type levels of sensitivity to MMS indicating that the myc-tagged protein was functional (Figures S3 and S4). We then introduced this tagged protein in the I-SceI strain, and performed ChIP experiments to test the binding of *RTT103* to DSBs (shown schematically in Figure 8a). I-SceI enzyme was induced for 3 hours and then proteins were cross-linked with formaldehyde and immunoprecipitated using anti-myc antibodies. We used Southern blots to confirm that the 3 hour induction produced endonuclease digests as expected (Figure S5). PCR primers were designed to amplify the site of damage, and 0.2 kb, 0.5 kb, 1 kb, 2 kb, and 3 kb away from the site of scission. *SPS2* on chromosome IV and a telomere-proximal site on chromosome VI were chosen as negative controls. Additionally, 3′ ends of two genes, *PMA1* and *ADH1*, where Rtt103 was shown to be enriched were also tested. As shown in Figure 8b, except for 3′ ends of *PMA1* and *ADH1*, where Rtt103 was expected to be enriched, there was no significant association of Rtt103 with any of the other regions tested when the endonuclease was not induced (blue bars). However, upon induction, the association of Rtt103 went up more than 5-fold only in the vicinity of the cut site (red bars). *SPS2*, telomere regions, regions further away from the cut site, *PMA1* and *ADH1* did not show any increased association upon endonuclease induction, suggesting that Rtt103 protein was binding specifically to regions of damage. We also carried out ChIP experiments at the same time for Yku80myc and confirmed that association of Yku80 with damaged sites was as expected (Fig. S6). These data strongly indicate that Rtt103 functions through association at the site of damaged DNA.

## Discussion

Telomeres are specialized ends of the chromosomes consisting of tandem repetitive sequences and proteins that bind to these sequences. Telomeres protect chromosome ends from fusion and prevent the natural chromosome ends from being recognized as DSBs by the DNA repair apparatus. Proteins involved in DSB repair, Yku70/80p, are localized to the telomeres and are critical for protection of chromosome ends. In an effort to understand the mechanisms through which this heterodimer protects chromosome ends, we isolated suppressors of *yku70*Δ. In the process we discovered an unanticipated role for transcription termination factor, Rtt103, in maintaining genome integrity.

Rtt103 is an abundant nuclear protein, however no clear function had been demonstrated previously. Our work for the first time connects *RTT103* to recovery from DSBs. We show that *RTT103* is essential for survival when genome integrity is compromised. Rtt103 also associates with sites of DNA damage and is likely to play a direct role in maintenance of genome integrity. Rtt103 contains a carboxyl-terminal domain interacting domain (CID) or RPR. This domain interacts with the conserved C-terminal domain of RNA polymerase II and proteins are recruited via this interaction to the actively transcribed chromatin [35]. *RTT103* is not essential; but it is synthetically lethal with several RNA 3′-end processing factors including *RAI1*, *CTK1* and *REF2*. Based on the interactions and the preferential association of Rtt103 with 3′-ends of genes, it is proposed that Rtt103 facilitates termination of transcription along with Rat1 and Rai1 proteins. *rtt103*Δ do not show obvious defects in termination [27].

Maintenance of genome integrity is essential for survival of cells and multiple pathways contribute to this process. Recently, evidence has accumulated to indicate the critical role of 3′-end processing in responding to damage [18]. A general response to UV treatment is the reduction of poly A+ mRNA [36] and 3′-end processing is also affected. CstF-50 is a component of CstF (cleavage stimulation factor), which is an essential polyadenylation factor and interacts with the CTD domain of RNAP-II to promote RNA processing. Upon UV-induced damage, it also interacts with BARD1 (BRCA1 associated RING domain protein) and through its association with BARD1 and BRCAI, promotes the inhibition of 3′-end processing. Thus CstF may coordinate the RNA processing activity with DDR. This work also shows that CstF-50, RNAP-II, BARD1, and BRCA1 associated with the sites of repaired DNA [18]. Sen1 helicase has recently been shown to be important in maintaining genome integrity [19]. *SEN1* prevents transcription-associated genome instability by preventing R-loop formation. R-loops are thought to form during transcription between the denatured DNA and the nascent transcript. These loops induce recombination leading to genome instability. The transcription termination mutant *rat1-1* did not show transcription-dependent recombination, suggesting that defect in transcription termination *per se* is unlikely to be the cause for such recombination defects. This is similar to our observation that *rat1-1* and *rai1* are not as sensitive to MMS as *rtt103*. Another intriguing link between RNA processing and genome stability is indicated by the consistent co-purification of KU70/80 and DNAPK during isolation of the human pre-RNA processing complex [37].

Our data support a more direct role for *RTT103* in responding to DNA breaks. Firstly, we see sensitivity of *rtt103*Δ to MMS and DNA DSBs, but not to UV radiation. As transcriptional responses are seen for all the three types of genome insults described here, a general transcriptional defect is likely to affect all types of repair. However, it still remains formally possible that transcripts specifically involved in these responses are affected in *rtt103*Δ. This possibility could be addressed by studying genome-wide transcriptional response to different DNA damaging agents in *rtt103*Δ. Secondly, we do not see sensitivity to DNA damage in *rai1* and *rat1-1* mutants that have even stronger termination defects. Thirdly, Rtt103 does not show the same types of genome instability or epistatic relationships with DNA repair pathways as those associated with mutations in *sen1* helicase and other RNA processing mutants which promote genome stability through prevention of R loops. Lastly, our studies clearly show over 5-fold enrichment of Rtt103 protein at the sites of DNA damage upon induction of damage. This strongly suggests that Rtt103 protein functions at the sites of damage.

There is some indirect evidence from various genome-wide studies to suggest that *RTT103* might be involved in damage response and other chromosomal functions since Rtt103 interacts genetically with a plethora of genes involved in chromatin or genome stability [38] For example, *rtt103*Δ show increased Rad52 foci, a mark of increased spontaneous damage [8]. These could be due to spontaneous DSBs in the unperturbed cells. *rtt103* is synthetically sick with *dna2*, a mutant that is sensitive to multiple forms of DNA damage [39]. *rtt103* was also found to produce growth defects in synthetic combinations with condensins [40] and *rtt103* is synthetically sick with *orc2-1* and *orc5-1* [41]. In all these networks neither *rat1* nor *rai1* appear. Given that *RAT1* is essential and may have been missed in these screens, if termination defects were the primary reason for picking up *rtt103*, *rai1* is likely to have appeared in a few of these screens. This suggests that *RTT103* may have an additional role in protecting genome integrity.

As Rtt103 has been isolated with transcription termination complexes and our studies show that it is essential for DNA damage response, we suggest a hitherto unidentified role for this machinery in genome stability. It is possible that termination complexes become associated with DNA damage sites to disengage RNA polymerase from the damaged sites in order to prevent synthesis of aberrant transcripts. Isolation of interacting complexes of Rtt103 in the presence of damaged DNA might indicate mechanistic basis for this function. Direct testing of other proteins in the termination complex for binding to the damaged sites will also be informative. In sum our results imply an essential role for *RTT103* in promoting genome stability and further investigations will be required to understand the mechanistic basis of this contribution.

## Supporting Information

Figure S1 **Expression of several DNA damage responsive genes is similar in WT and** ***rtt103***Δ**.** RNA was isolated from WT and *rtt103*Δ and northern blots were done to check the expression of *RNR2*, *RAD51*, *DUN1* and *PLM2*. *rtt103*Δ show similar pattern of upregulation of *RNR2* and *DUN1* as seen in WT cells. Upper panels show the northern blots probed with the indicated probes while the lower panel shows the corresponding agarose gel stained with ethidium bromide as loading control.
(TIF)

Figure S2 **Epistasis analysis of** ***rtt103*** **with** ***rad52 and yku70*** **.** WT and strains carrying deletions of *rtt103*, *rad52*, *rtt103rad52* and *rtt103yku70* were tested for sensitivity to MMS. *rtt103*Δ does not affect the sensitivity of *rad53* to DNA damage induced by MMS. *rtt103*Δ exacerbates the sensitivity of *yku70* to MMS.
(TIF)

Figure S3 **Rtt103 is nuclear localized.** The *RTT103* Myc strain was stained with anti-rabbit myc antibody. Rtt103p is localized in the nucleus. The nucleus is marked by both DAPI staining and antibody to a nuclear pore complex protein, Nsp1.
(TIF)

Figure S4 ***RTT103*** **13Myc is functional.** The indicated strains were spotted on YPD containing various concentrations of MMS. 5 µl of 10 fold serial dilutions of the strains were spotted and incubated at 30°C for 2 days. *rtt103* and *yku70* are sensitive to MMS where as *RTT103* 13Myc is not MMS sensitive indicating that the C-terminal tagging did not affect the function of *rtt103*.
(TIF)

Figure S5 **Induction of I-Sce1 cut by galactose.** (**a**) Schematic representation of the *URA3* region showing I-SceI site, the region used as probe and the products that are produced after the I-Sce1 cut. (**b**) Southern blot showing the I-SceI cut. The genomic DNA was isolated from the uninduced, 1 hr, 3 hr and overnight induced strain (KRY448) with galactose. The *Bgl*II digested DNA was run on an agarose gel, transferred on to membrane and then probed. Uninduced gives a product of ∼2500 bp and the I-SceI cut (induced) gives an uncut product of ∼2500 bp and cut products of ∼1500 and 1000 bp.
(TIF)

Figure S6 **Binding of Yku80p to the site of DNA damage: Binding of Yku80p to the site of DNA damage.** (**a**) Schematic diagram of region in and around *URA3* with two flanking I-Sce1 sites. Bars (1–8) represent the regions up to 1 kb away from Sce1 sites, at which Rtt103p binding is checked. (**b**) ChIP experiment to show Yku80p binding at the site of damage. Yku80-13Myc strain (KRY447) was grown in galactose medium to induce the expression of I-SceI enzyme for three hours and then cross-linked with formaldehyde and immunoprecipitated using anti-myc antibody. RT PCR was done to check the fold induction of Yku80p at the sites indicated. The numbers on the x-axis indicate the binding site of primers which is shown in the schematic diagram. The numbers on the y-axis indicate the fold induction of Yku80p compared to the internal *SPS2* gene (9) which is a house keeping gene. Error bars denote SD.
(TIF)
