# Supplementary figures and images for: Yeast Transcription Termination Factor Rtt103 Functions in DNA Damage Response
Source: PLoS One. 2012 Feb 15;7(2):e31288. doi: 10.1371/journal.pone.0031288 (PMC3280293; doi:10.1371/journal.pone.0031288)

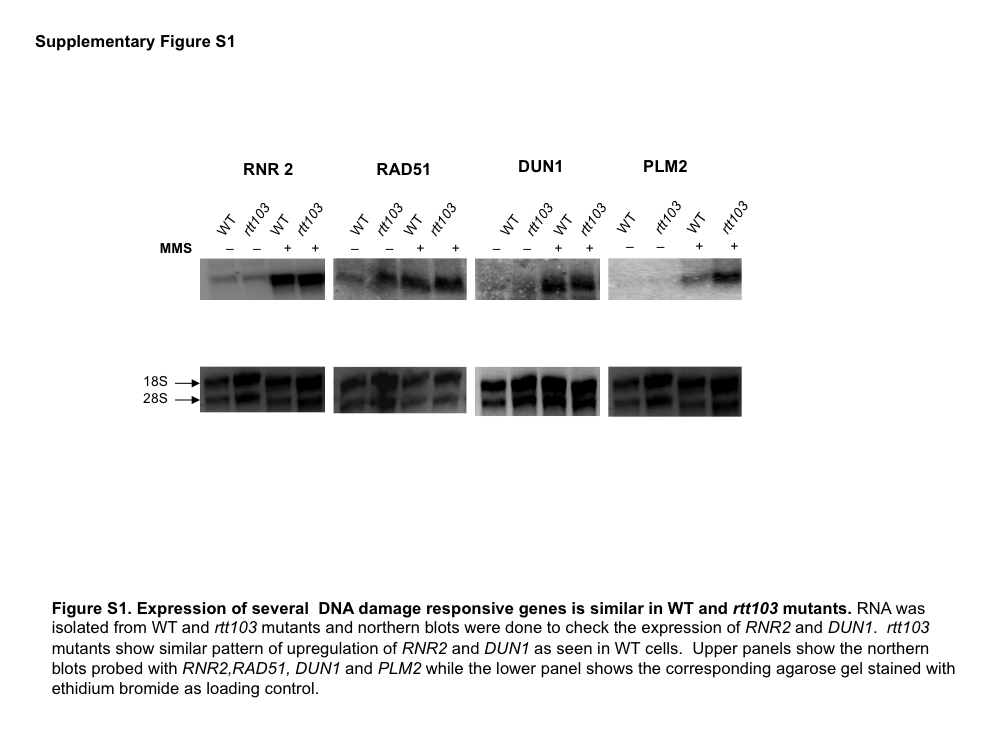

Supplement: Figure S1 — Expression of several DNA damage responsive genes is similar in WT and rtt103Δ. RNA was isolated from WT and rtt103Δ and northern blots were done to check the expression of RNR2, RAD51, DUN1 and PLM2. rtt103Δ show similar pattern of upregulation of RNR2 and DUN1 as seen in WT cells. Upper panels show the northern blots probed with the indicated probes while the lower panel shows the corresponding agarose gel stained with ethidium bromide as loading control. (TIF) [file pone.0031288.s001.tif]

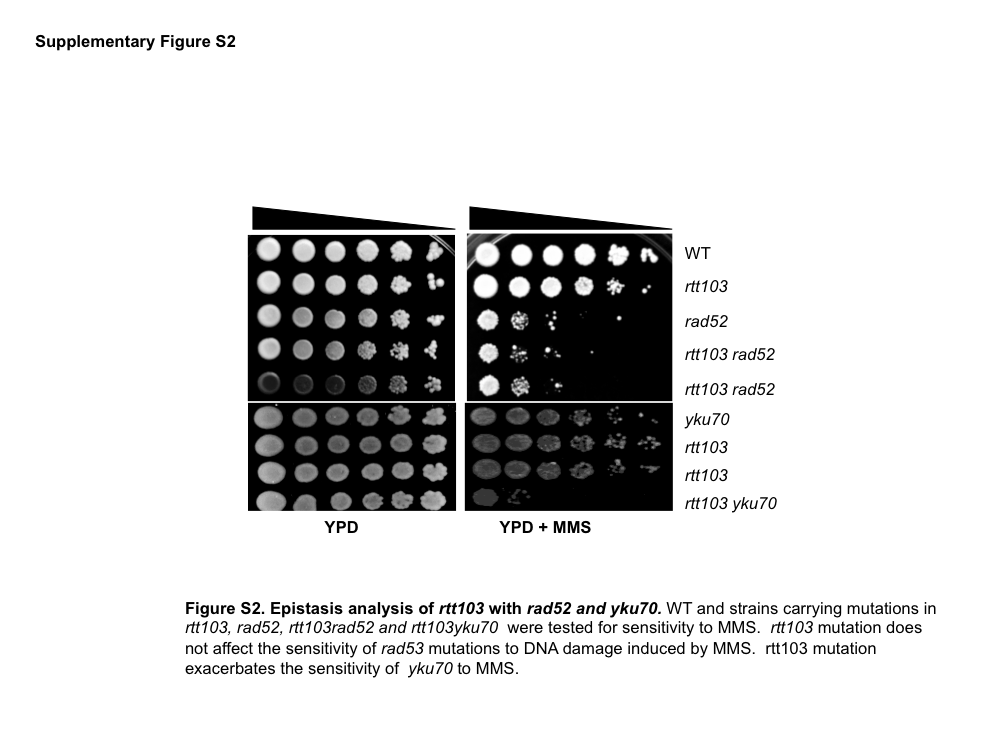

Supplement: Figure S2 — Epistasis analysis of rtt103 with rad52 and yku70 . WT and strains carrying deletions of rtt103, rad52, rtt103rad52 and rtt103yku70 were tested for sensitivity to MMS. rtt103Δ does not affect the sensitivity of rad53 to DNA damage induced by MMS. rtt103Δ exacerbates the sensitivity of yku70 to MMS. (TIF) [file pone.0031288.s002.tif]

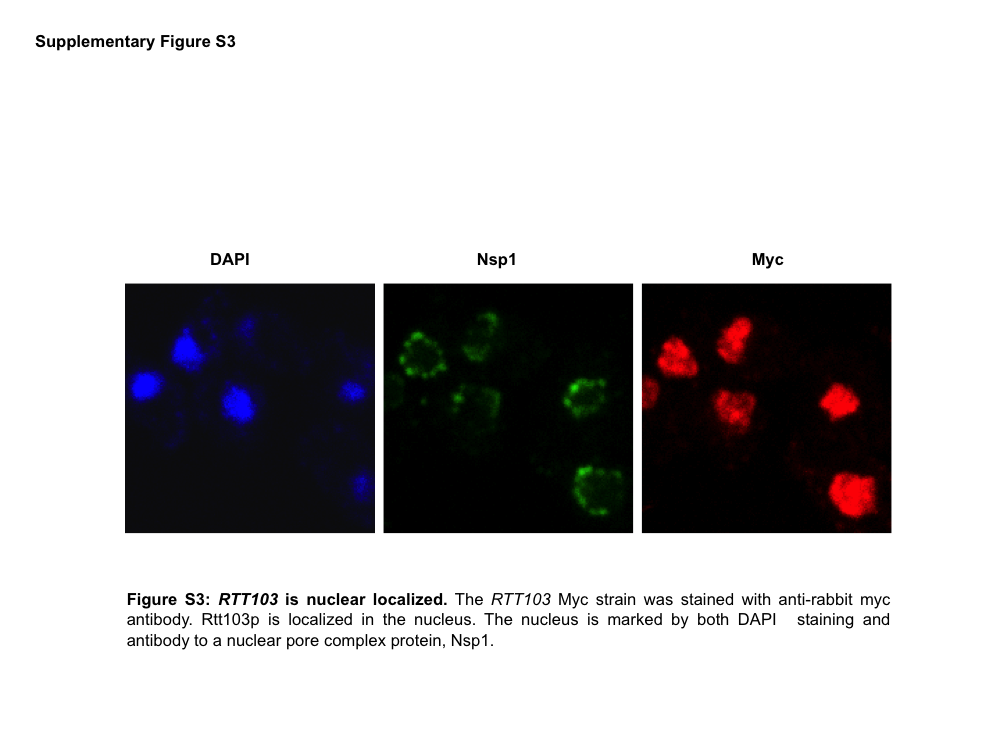

Supplement: Figure S3 — Rtt103 is nuclear localized. The RTT103 Myc strain was stained with anti-rabbit myc antibody. Rtt103p is localized in the nucleus. The nucleus is marked by both DAPI staining and antibody to a nuclear pore complex protein, Nsp1. (TIF) [file pone.0031288.s003.tif]

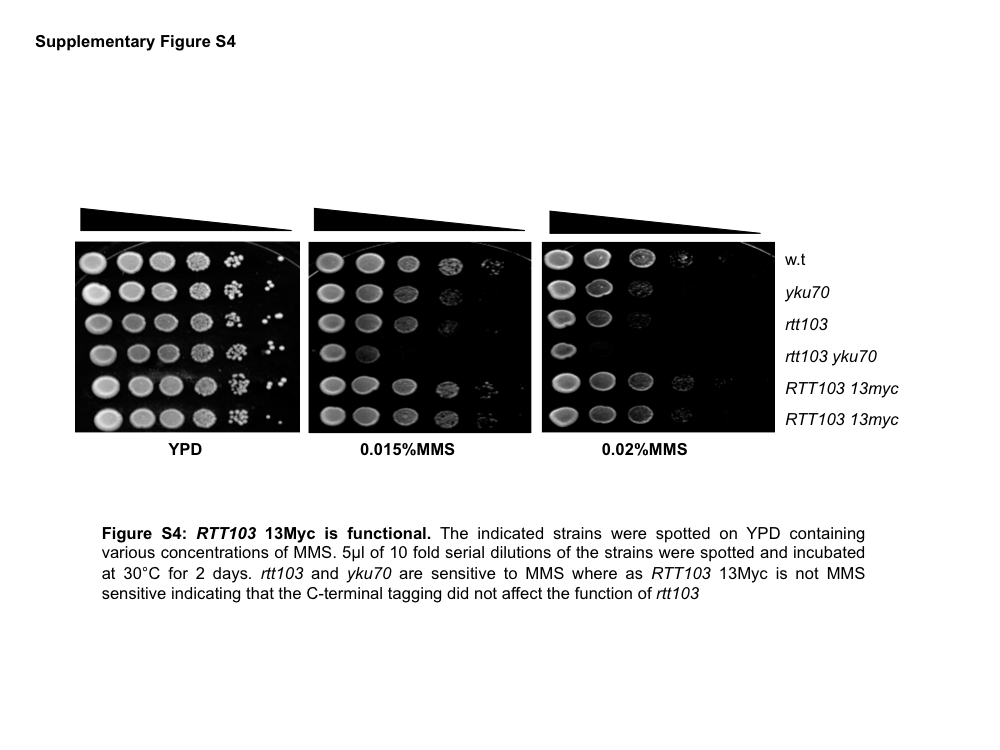

Supplement: Figure S4 — RTT103 13Myc is functional. The indicated strains were spotted on YPD containing various concentrations of MMS. 5 µl of 10 fold serial dilutions of the strains were spotted and incubated at 30°C for 2 days. rtt103 and yku70 are sensitive to MMS where as RTT103 13Myc is not MMS sensitive indicating that the C-terminal tagging did not affect the function of rtt103. (TIF) [file pone.0031288.s004.tif]

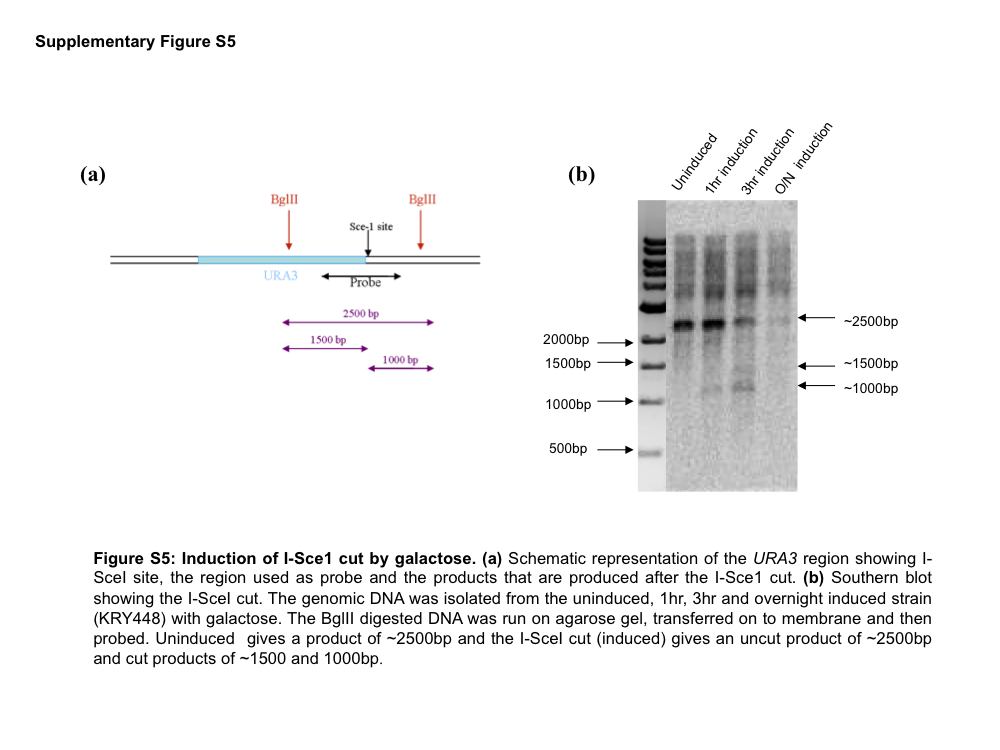

Supplement: Figure S5 — Induction of I-Sce1 cut by galactose. (a) Schematic representation of the URA3 region showing I-SceI site, the region used as probe and the products that are produced after the I-Sce1 cut. (b) Southern blot showing the I-SceI cut. The genomic DNA was isolated from the uninduced, 1 hr, 3 hr and overnight induced strain (KRY448) with galactose. The BglII digested DNA was run on an agarose gel, transferred on to membrane and then probed. Uninduced gives a product of ∼2500 bp and the I-SceI cut (induced) gives an uncut product of ∼2500 bp and cut products of ∼1500 and 1000 bp. (TIF) [file pone.0031288.s005.tif]

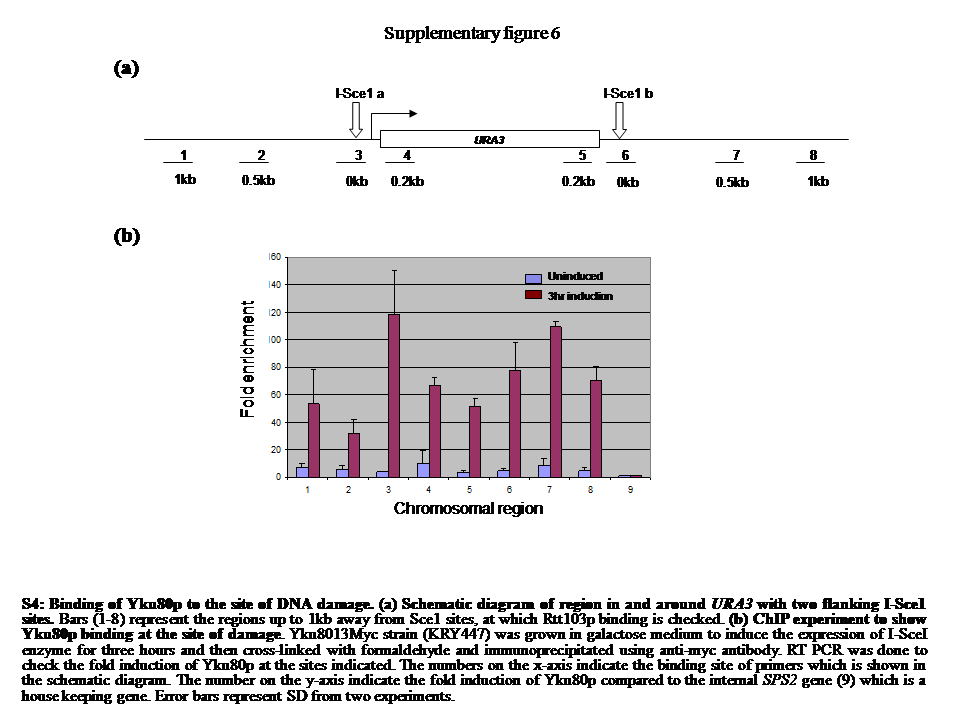

Supplement: Figure S6 — Binding of Yku80p to the site of DNA damage: Binding of Yku80p to the site of DNA damage. (a) Schematic diagram of region in and around URA3 with two flanking I-Sce1 sites. Bars (1–8) represent the regions up to 1 kb away from Sce1 sites, at which Rtt103p binding is checked. (b) ChIP experiment to show Yku80p binding at the site of damage. Yku80-13Myc strain (KRY447) was grown in galactose medium to induce the expression of I-SceI enzyme for three hours and then cross-linked with formaldehyde and immunoprecipitated using anti-myc antibody. RT PCR was done to check the fold induction of Yku80p at the sites indicated. The numbers on the x-axis indicate the binding site of primers which is shown in the schematic diagram. The numbers on the y-axis indicate the fold induction of Yku80p compared to the internal SPS2 gene (9) which is a house keeping gene. Error bars denote SD. (TIF) [file pone.0031288.s006.tif]
